# Costs and benefits of multiple resistance to insecticides for *Culex quinquefasciatus *mosquitoes

**DOI:** 10.1186/1471-2148-8-104

**Published:** 2008-04-08

**Authors:** Claire Berticat, Julien Bonnet, Stéphane Duchon, Philip Agnew, Mylène Weill, Vincent Corbel

**Affiliations:** 1 Institut des Sciences de l'Evolution, UM2, CNRS ; Equipe Génétique de l'Adaptation, Université Montpellier 2, C.C. 065, 34095 Montpellier, France; 2 Laboratoire de Lutte contre les Insectes Nuisibles, Institut de Recherche pour le Développement, 911 Avenue Agropolis (BP 64501), 34394 Montpellier, France; 3 Génétique et Evolution des Maladies Infectieuses, UMR 2724 CNRS-IRD, 911 Avenue Agropolis (BP 64501), 34394 Montpellier, France

## Abstract

**Background:**

The evolutionary dynamics of xenobiotic resistance depends on how resistance mutations influence the fitness of their bearers, both in the presence and absence of xenobiotic selection pressure. In cases of multiple resistance, these dynamics will also depend on how individual resistance mutations interact with one another, and on the xenobiotics applied against them. We compared *Culex quinquefasciatus *mosquitoes harbouring two resistance alleles *ace-1*^*R *^and *Kdr*^*R *^(conferring resistance to carbamate and pyrethroid insecticides, respectively) to mosquitoes bearing only one of the alleles, or neither allele. Comparisons were made in environments where both, only one, or neither type of insecticide was present.

**Results:**

Each resistance allele was associated with fitness costs (survival to adulthood) in an insecticide-free environment, with the costs of *ace-1*^*R *^being greater than for *Kdr*^*R*^. However, there was a notable interaction in that the costs of harbouring both alleles were significantly less than for harbouring *ace-1*^*R *^alone. The two insecticides combined in an additive, synergistic and antagonistic manner depending on a mosquito's resistance status, but were not predictable based on the presence/absence of either, or both mutations.

**Conclusion:**

Insecticide resistance mutations interacted to positively or negatively influence a mosquito's fitness, both in the presence or absence of insecticides. In particular, the presence of the *Kdr*^*R *^mutation compensated for the costs of the *ace-1*^*R *^mutation in an insecticide-free environment, suggesting the strength of selection in untreated areas would be less against mosquitoes resistant to both insecticides than for those resistant to carbamates alone. Additional interactions suggest the dynamics of resistance will be difficult to predict in populations where multiple resistance mutations are present or that are subject to treatment by different xenobiotics.

## Background

Resistance to xenobiotics (antibiotics, insecticides, herbicides, etc...) is a problem limiting our ability to control organisms of a medical or economic importance. Multiple resistance, where organisms harbour separate mutations conferring resistance against more than one type of xenobiotic, poses an even greater problem. Furthermore, the number of cases documenting multiple resistance are increasing in frequency and involve a broad range of target organisms, including; viruses [1], bacteria [2], fungi [3], plants [4], and insects [5].

While little can prevent the emergence of resistance, short of stopping xenobiotic use, it should be possible to manage resistance in target populations by regulating how, when, and which xenobiotics are used. To do so, it is important to understand the evolutionary dynamics of resistance in both the presence and absence of xenobiotic selection pressure. This is because the extent to which a target population experiences these two types of environment will have a strong influence on the frequency of resistance and the rate at which it changes. For example, migration of susceptible and resistant individuals between treated and untreated areas influences spatial and temporal variations in the frequency of insecticide resistance alleles in populations of the peach-potato aphid *Myzus persicae*, [6-8] and *Culex pipiens *mosquitoes [9,10]. In each case, resistance alleles provide a fitness benefit to their bearers in the presence of a particular insecticide and can rapidly increase in frequency within populations. However in the absence of insecticide pressure, these resistance alleles are often associated with fitness costs that lead to them decreasing in frequency as resistant individuals are out-competed by susceptible rivals. For example, resistant individuals have been found to have lower mating success [11], be more susceptible to natural enemies [12,13], or more prone to mortality during over-wintering [14]. However, there are some counter-examples where resistant individuals show a gain in their fitness relative to susceptible rivals in untreated areas. The traits involved include greater mating success [15,16], and being less susceptible to the costs of natural enemies [12]. Thus it is important to identify traits that might be affected by resistance alleles and contexts in which they may influence fitness.

Far less information is available to estimate the dynamics of multiple resistance where resistance to more than one type of xenobiotic is conferred by the presence of resistance alleles at more than one locus. This situation is potentially more complex as resistance alleles could interact with one another to affect the fitness of their bearers, and these interactions could depend on the environmental context determined by the presence/absence of one or more xenobiotic. Indeed, there is evidence from natural populations of mosquitoes that the frequency of doubly-resistant individuals in untreated areas depart from Hardy-Weinberg expectations, indicating that alleles at different loci in these populations do interact with one another to affect the fitness of their bearers [17,18], and available data suggests a similar pattern may be occurring in aphids [6]. In addition, it is known that the efficacy of a xenobiotic can depend on the presence of other xenobiotics in the environment, such that the combined activity of two xenobiotics is greater (synergistic) or less (antagonistic) than expected based on the sum of activity for each compound separately [19]. Furthermore, whether two xenobiotics act synergistically or antagonistically can depend on the presence or absence of a particular resistance allele in the target individual, as has been found for carbamate and pyrethroid activity against *Culex quinquefasciatus *mosquitoes with or without the *ace-1*^*R *^allele at the *ace-1 *locus [20,21]. Hence, the evolutionary dynamics of multiple resistance are open to modification by interactions among alleles at different loci within the organism, interactions involving alleles and toxins in the environment, and interactions among toxins in the environment. Identifying these interactions and how they influence the fitness of multiply resistant organisms could provide useful information for operational strategies of resistance management and throw light on the physiological processes underlying them.

In the following article, we investigated the fitness effects of two insecticide resistance alleles, *ace-1*^*R *^and *Kdr*^*R*^, in four strains of *C. quinquefasciatus *that differed in harbouring only one resistance allele (either *ace-1*^*R *^or *Kdr*^*R*^), both alleles, or neither allele (Table 1). These mutations confer resistance to carbamate and pyrethroid insecticides, respectively. As an important vector of West Nile virus in North America [22], and a major vector of bancroftian filariasis in Africa [23], *C. quinquefasciatus *is frequently subject to control by insecticides, including the two mentioned above. The two resistance alleles involved in this study are present in natural mosquito populations, and some populations harbour both mutations [24,25]. Thus, multiple resistance is a potential problem in these populations.

**Table 1:** Description of strain resistance genotypes. Genotypic description of each strain at the *ace-1 *and *Kdr *loci. Insecticide resistance alleles are in bold. All strains are homozygous at both loci.

Strains^a^	*ace-1 *allele	*Kdr *allele
SLAB	*ace-1*^*S*^	*Kdr*^*S*^
SR	***ace-1*^*R*^**	*Kdr*^*S*^
BC	*ace-1*^*S*^	***Kdr*^*R*^**
BCSR	***ace-1*^*R*^**	***Kdr*^*R*^**

^a ^Insecticide resistance alleles are in bold. All strains are homozygous at both loci

Fitness estimates for resistance alleles, including any potential interactions among them, can vary according to the genetic background in which they are expressed [26,27], and on the environmental conditions in which they are measured [28,29]. To control for the first factor, the two resistance alleles involved in this study were separately backcrossed into a common and susceptible genetic background provided by the SLAB strain of *C. quinquefasciatus *[30]. The doubly-resistant strain was derived by backcrossing the two singly-resistant strains (see Methods for full details). Hence, differences between strains could be directly attributed to the expression of resistance alleles in a standard genetic environment. Measurements of fitness were performed in standardised laboratory conditions that differed only in the presence/absence of one or both insecticides, thereby minimising other potentially confounding sources of genotype-by-environment interactions.

## Results

### Fitness costs associated with resistance alleles

**Figure 1:**
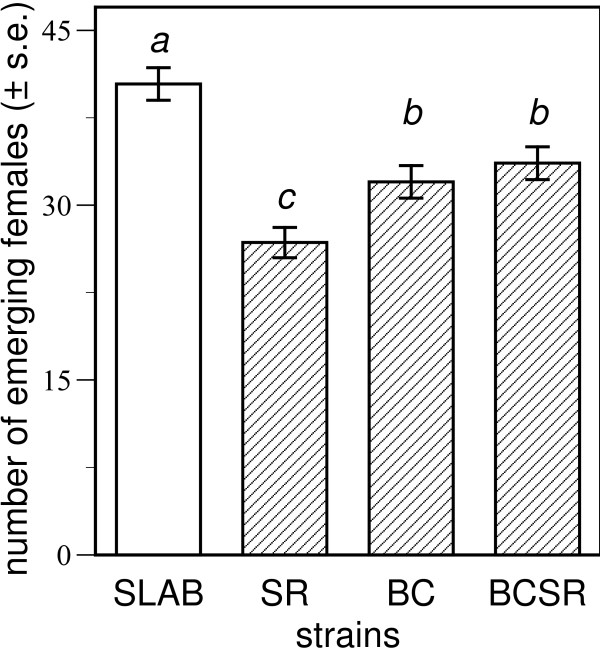
**Costs of resistance in an insecticide-free environment**. The number of female mosquitoes emerging in an insecticide-free environment. Insecticide susceptible strain SLAB is shown in an open column and the three resistant strains are shown in hatched columns. In two replicate experiments, there were nine or ten pots initially containing 100 larvae for each of the four strains. Different letters above columns indicate significant differences (*p *< 0.05) in female emergence as found by a Tukey-Kramer test (see text for details).

There were costs associated with insecticide resistance in an insecticide-free environment (Figure 1). Strains differed in the average probability of female emergence (minimal model; Strain, *F *[3,74] = 17.226, *p *< 0.001, *r*^2 ^= 0.41), with a Tukey-Kramer HSD test finding emergence from the susceptible SLAB strain to be significantly greater than for the three resistant strains. The same test also found the emergence of strains BC and BCSR to not be different, and that both were significantly greater than for strain SR. In other words, doubly-resistant females from strain BCSR, harbouring both *ace-1*^*R *^and *Kdr*^*R *^alleles, were significantly more likely to reach adulthood than singly-resistant females of strain SR with only the *ace-1*^*R *^allele.

### Insecticide interactions

**Figure 2:**
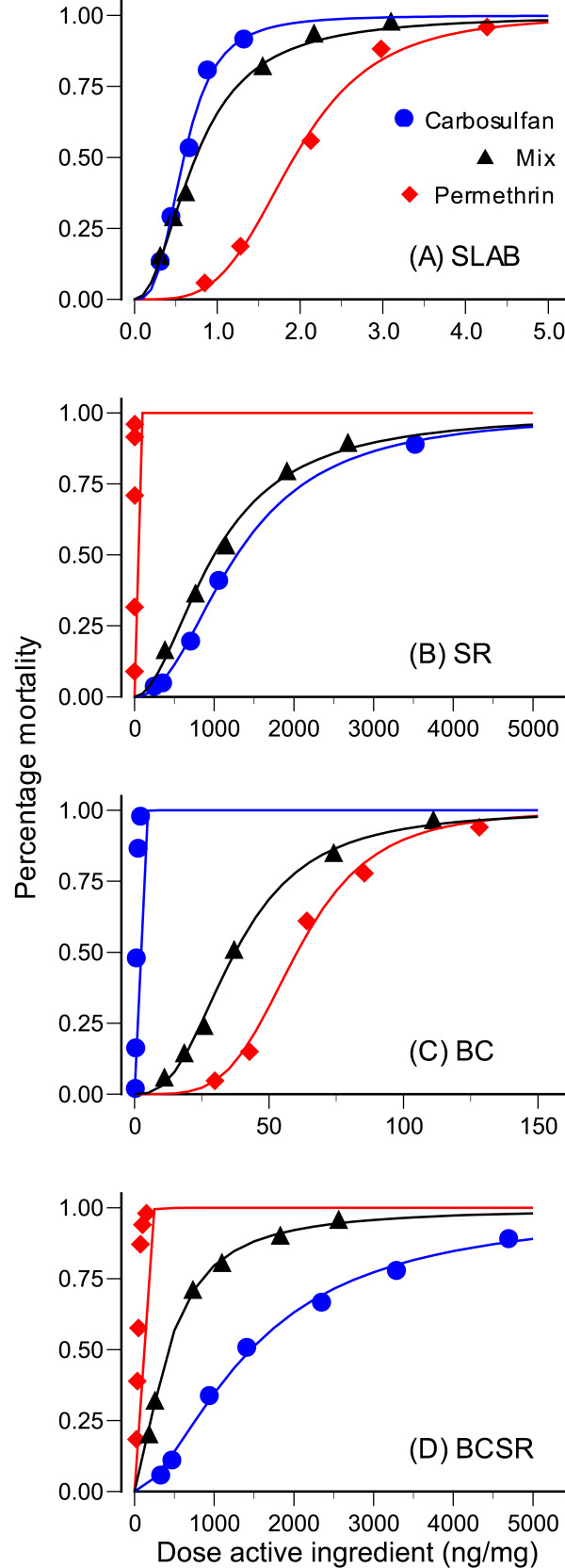
**Dose-effect curves for insecticide activity**. Dose-effect curves of permethrin, carbosulfan and their mixture on the four strains of mosquito.

The dose-effect curves for permethrin, carbosulfan and their mixtures on the mortality of adult female mosquitoes of each strain (SLAB, SR, BC and BCSR) are shown in Figure 2. For each strain, the dose-mortality relationships were sigmoidal. Mortality in control batches never exceeded 10%. Median lethal doses for these relationships are listed in Table 2.

**Table 2:** Median-lethal doses used. Median-lethal doses ng/mg (± 95% confidence intervals) for mosquito strains tested with different insecticides and their mixture.

Strain	SLAB		SR		BC		BCSR	
insecticide								
Permethrin (P)	1.85	(1.75–1.95)	1.80	(1.71–1.89)	62.65	(59.38–66.10)	41.47	(39.56–43.47)
Carbosulfan (C)	0.59	(0.52–0.66)	1264.00	(1166–1370)	0.78	(0.68–0.90)	1446.00	(1330–1572)
Mix	0.99	(0.90–1.08)	982.00	(884–1084)	37.56	(34.97–40.39)	438.00	(386–492)
mix ratio P:C	3:1		1:750		85:1		1:35	
regression coefficient^a ^± (s.e.)								
Permethrin	3.76^a^	(0.18)	3.48^a^	(0.15)	4.04^a^	(0.22)	3.05^a^	(0.10)
Carbosulfan	3.01^b^	(0.16)	2.17^b^	(0.07)	3.73^a^	(0.31)	1.89^b^	(0.07)
Mix	2.95^b^	(0.22)	2.69^c^	(0.22)	3.53^a^	(0.20)	2.15^c^	(0.15)

^a ^estimates within a column that differ in super-script letter are significantly different (*p *< 0.05)

**Figure 3:**
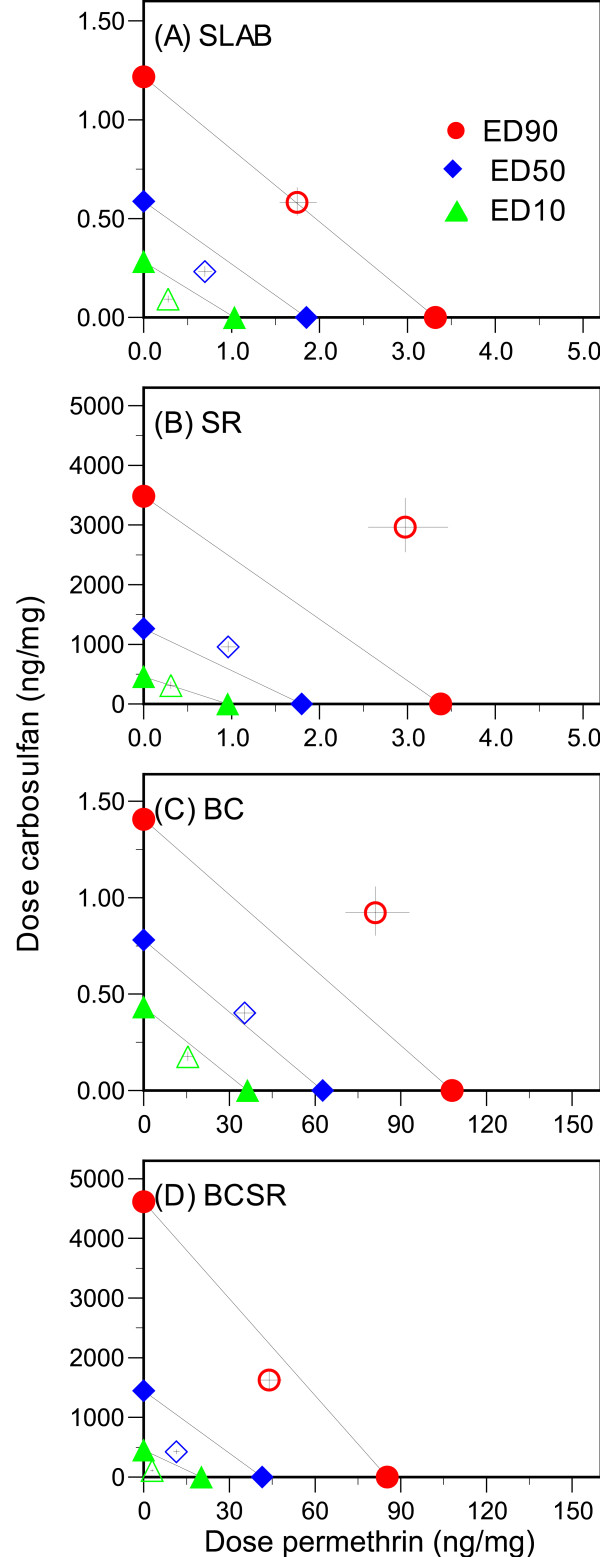
**Isobolograms of insecticide activity**. Diagonal lines connect doses of equally effective activity for each insecticide when applied alone. Open symbols are for the amount of each insecticide required for the same effect when the two are mixed together at a ratio based on their median-effect doses (± 95% confidence intervals). Points above the matching line indicate antagonistic activity of the insecticides, close to the line indicates additivity of action, and those below the line indicate synergistic activity.

Figure 3 illustrates whether there was additive, synergistic or antagonistic activity of mixtures containing both insecticides. Diagonal lines connect doses of permethrin on the *x*-axis with doses of carbosulfan on the *y*-axis where each insecticide was equally efficient at killing mosquitoes when applied alone (isoboles ED_10_, ED_50 _and ED_90 _are shown). The position of the matching data points relative to these lines indicates how much of each insecticide was required to achieve the same effect when insecticides were mixed at a ratio determined by their median-effect doses: data points above the line indicate antagonistic activity of the two insecticides, points close to the line indicate additive activity, while those below the line reveal synergistic activity of the two compounds.

The susceptible strain SLAB showed, a limited degree of synergy in activity, especially when the treatment doses applied induced <80% mortality. That is, the mixture of insecticides was more potent or effective than would be expected if each had acted in an independent and additive manner. However, at higher doses inducing >80% mortality, the two compounds acted in an additive fashion (Figure 3A). In contrast, the two insecticides acted antagonistically when applied to the carbamate-resistant strain SR, especially once >25% of the mosquitoes were killed during assays (Figure 3B). Hence, the combination of insecticides was less efficient than would be expected had each acted separately in an additive fashion. A simple additive effect was found for the pyrethroid-resistant BC strain in the low to medium range of insecticide activity, while at high doses antagonistic behaviour was observed (Figure 3C). Finally, the doubly resistant strain BCSR, showed striking synergism between the pyrethroid and carbamate activity (Figure 3D) indicating the mix of insecticides was more efficient at killing mosquitoes than if each had acted additively.

## Discussion

Evolutionary theory predicts that mutations of large phenotypic effect, such as resistance mutations, are likely to be costly to their bearers in environments where they were not selected [31,32]. We found costs associated with both resistance mutations in an insecticide-free environment (Figure 1). Significantly fewer females emerged as adults from resistant strains SR and BC in comparison to the susceptible control strain SLAB. These costs could be associated with how the resistance mutations influence the nervous system. In strain SR, the *ace-1*^*R *^mutation causes hyper-activity of nervous systems due to a modified acetylcholinesterase (AChE) that is less efficient at catalysing the breakdown of acetylcholine (ACh) bound to nicotinic receptors at the post-synaptic membrane [33]. In the field, fewer homozygotes of *ace-1*^*R *^were found than would be expected from the Hardy-Weinberg ratio, indicating reduced viability of these homozygotes [17,18]. Recent findings from studies on *Drosophila melanogaster *suggest these costs could directly arise from a reduced stability of the enzyme due to changes in its deacetylation [34]. In contrast, Lee et al. observed changes in the pharmacological and biophysical properties of sodium channels in *Heliothis virescens *moths with pyrethroid resistance due to a *Kdr *mutation [35]. These changes were found to cause sluggish neural activity in the absence of pyrethroids and were characterized by decreased cellular and behavioural excitability of sodium channels [35]. The *Kdr *mutation enhances closed-state inactivation of nerves, meaning that more stimulation is required before nerves fire and release ACh into the synaptic cleft in comparison with susceptible individuals [36].

The fitness of the BCSR strain harbouring both *ace-1*^*R *^and *Kdr*^*R *^was of particular interest. Reduced female emergence, relative to the susceptible SLAB strain, showed there were costs of resistance for this strain. However, the costs of harbouring both resistance alleles were significantly less than for strain SR only harbouring the *ace-1*^*R *^mutation (Figure 1). We speculate that the relatively greater costs associated with a reduced efficiency of ACh degradation at the post-synaptic terminal (due to *ace-1*^*R*^) could be partially compensated for by a more sluggish release of ACh at the pre-synaptic terminal (due to *Kdr*^*R*^). This hypothesis of how the two alleles interact at the functional level could be tested by electrophysiological examination of the synaptic activity of insects harbouring only one resistance allele (either *ace-1*^*R *^or *Kdr*^*R*^), both alleles, or neither allele.

The reduced costs experienced by strain BCSR relative to strain SR in an insecticide-free environment are noteworthy as they illustrate that one resistance allele can act as a compensatory mutation for another resistance allele, and both can be mutations of large effect. Our results would predict the addition of *Kdr*^*R*^, by mutation or recombination, to a genotype with *ace-1*^*R *^already present would reduce the strength of selection against *ace-1*^*R *^in an insecticide-free environment. Thus not only would the strength of selection against *ace-1*^*R *^be reduced in untreated populations, but this allele would remain for longer in untreated populations in mosquitoes with both resistance alleles and that were resistant to both carbamate and pyrethroid insecticides, rather than in mosquitoes only resistant to carbamates. Although this prediction can only strictly be applied to *C. quinquefasciatus *mosquitoes of the SLAB strain, the role of compensatory activity among resistance alleles on the evolution and control of multiple resistance warrants general investigation, as this experiment provides evidence that it can occur.

We also found evidence that resistance alleles interacted with one another in the presence of both insecticides. When mosquitoes were tested against mixtures of permethrin and carbosulfan, strain SLAB showed evidence for additive activity of the two insecticides at high doses (Figure 3A), while BC showed additive activity at low to medium doses (Figure 3C). In contrast, there was a synergy of activity at low doses when both insecticides were tested against SLAB (Figure 3A), but this effect was more striking for the doubly-resistant BCSR strain (Figure 3D). This latter result confirms previous studies involving the SLAB strain [18,20], where these studies showed both insecticides complemented each other as both cause ACh to accumulate in synaptic clefts. Furthermore, at sufficiently high concentrations, ACh simulates the activity of muscarinic receptors M2 at the pre-synaptic terminal [37]. Simulation of these receptors inhibits further release of ACh into the synaptic cleft. This enhanced negative feedback inhibition of ACh release effectively shuts down neural activity and leads to the insect's death.

There was synergism in insecticide activity when applied to BCSR, but antagonism for strain SR (Figure 3B). We cannot currently explain this result. Although the mode of action for pyrethroids and carbamates against susceptible insects have described in detail [38,39], it is less clear as to how they induce mortality when organisms harbour multiple resistance alleles. From a physiological point of view, point mutations in sodium channels and acetylcholinesterase are responsible for reduced insecticide sensitivity in several insect species [17,34,36]. Consequently, when the primary targets of insecticides become insensitive (as is the case for BCSR and SR), higher doses are required to achieve equivalent mortality and secondary target sites may be involved. In doing so, they add to the number of events influencing neuronal activity and the pathways by which insecticides can act and interact with one another, e.g., GABA receptor – ionophore complex or chloride channel and muscarinic receptors have been shown to be secondary target sites for pyrethroids and carbamates, respectively [39,40]. To our knowledge, the only publication investigating the functional costs of harbouring multiple resistance alleles was published by Bourguet et al. in 1997 [33]. In this article the authors showed AChE activity in the SR strain of *C. quinquefasciatus *was unaffected by the treatment of propoxur (a carbamate), even at doses of insecticides inducing 100% mortality. This lead to the suspicion that mortality of SR larvae was not due to AChE inhibition, but to the insecticide interacting with another target site, known as choline acetyltransferase, or ChAT, which is involved in ACh synthesis [41]. This hypothesis was supported by the behavioural abnormality of moribund larvae due to a lack of ACh in the synapse and the fact that *in vitro *inhibition of ChAT activity was caused by doses of propoxur inducing mortality of the SR strain [33].

A greater understanding of the mechanisms involved in what determines positive or negative interactions of insecticide activity will require further toxicological and electrophysiological studies involving single- and multiple-resistance alleles in appropriate environments where their effects can be directly compared and contrasted.

## Conclusion

Our results show the resistance alleles *ace-1*^*R *^and *Kdr*^*R *^interact with one another to influence the fitness of *C. quinquefasciatus *mosquitoes in a laboratory environment, and that they did so in both the presence and absence of insecticides. A particularly interesting, and potentially worrying, observation was that *Kdr*^*R *^could compensate for the costs of *ace-1*^*R *^in an insecticide-free environment. This would predict selection against *ace-1*^*R *^in untreated environments would be slower in mosquitoes resistant to both pyrethroid and carbamate insecticides, rather than just resistant to carbamates. More needs to be done to verify if this is an isolated interaction or if it more widely reflects how resistance alleles may interact. We also found a female mosquito's resistance status influenced whether mixtures of carbamate and pyrethroid insecticides had additive, synergistic or antagonistic activity. Overall these results suggest the evolutionary dynamics of resistance will be difficult to predict in populations where multiple resistance mutations are present or that are subject to treatment by different xenobiotics.

## Methods

### Mosquito strains

Four strains of *C. quinquefasciatus *were used; SLAB, SR, BC, and BCSR. They all share the same genetic background and cytoplasm, and only differ in their genotype at the *ace-1 *and *Kdr *loci. SLAB, the insecticide susceptible reference strain [30], is homozygous for susceptible alleles at both loci. SR is homozygous for the resistant allele *ace-1*^*R *^and for the susceptible allele *Kdr*^*S *^and was introgressed into the genome of SLAB through 14 repeated generations of backcrossing [11]. BC is homozygous for the susceptible allele *ace-1*^*S *^and for the resistant allele *Kdr*^*R*^; BCSR is homozygous for resistant alleles at both loci (Table 1).

The BC strain was derived from the B-KPER strain [20] whose genome was introgressed into that of SLAB through 13 repeated generations of backcrossing. At each generation, a discriminating dose of permethrin (3 mg l^-1^), a pyrethroid, was applied to select for resistant heterozygotes at the *Kdr *locus, and surviving females were crossed with SLAB males. During the last generation of backcrossing, surviving males were crossed with SLAB females in order to introduce the SLAB cytoplasm into the introgressed strains. Finally, homozygosity of the strains for these resistance alleles was verified by analysing parents using the molecular test of Weill *et al*.. [17] BCSR was derived from the cross between SR and BC strains. Homozygosity of this strain for resistant alleles at the *Kdr *and *ace-1 *loci was achieved by the F3 generation, as confirmed by the molecular test of Martinez-Torres *et al*. [42] and Weill *et al*. [17], respectively. To insure differences in resistance and life-history traits between the four strains were not due to maternal effects, each strain was reared for at least two generations in standard laboratory conditions without insecticide selection, before experiments began.

### Fitness costs of resistance alleles

We measured the cost of the *Kdr*^*R *^and *ace-1*^*R *^resistance alleles on the probability of female mosquitoes reaching adulthood. The oviposition of the four parental strains was synchronised and nine or ten groups of 100 first-instar larvae for each strain were transferred to pots containing 200 ml of tap water and 0.1 g of yeast. These pots were randomly distributed on the same table in a single room with temperature and light controlled (22° to 25°C, with a 12L:12D photoperiod). From, three days post-hatching, water and food were changed daily (food *ad libitum*) and the number and sex of emerging adults was recorded. This experiment was performed twice and involved a total of 3900 larvae in each experiment.

The number of adult females emerging from each pot was analysed and did not require transformation to meet requirements for parametric analysis. We focussed on females as only adult females were involved in the insecticide trials described below, and because female survival to adulthood is much more epidemiologically important than male survival. Female emergence was analysed with a fully factorial analysis of variance (ANOVA) with strain (SLAB, SR, BC, BCSR) as a fixed nominal factor and replicate experiment as a random nominal factor. Models were simplified when *F*-tests found no significant difference between full and reduced models. In each case, Shaprio-Wilk *W *goodness of fit tests found residuals were not significantly different from a normal distribution (analyses not shown). Analyses for the number of emerging males or total adult emergence gave similar results, and adult sex ratios did not depart from a 1:1 ratio (analyses not shown). Analyses were performed using JMP software (V. 5.1.2, SAS Institute 2004).

### Analysis of insecticide interactions

Topical applications were used to study the interactions between pyrethroids and carbamates because they allow an accurate estimation of the intrinsic toxicity of an insecticide by excluding all other effects linked to a mosquito's behaviour [43]. First, topical solutions were prepared by dissolving technical grades of permethrin (96% *cis:trans *ratio 25:75, Bayer CropScience, Villefranche-sur-Saone, France) and/or carbosulfan (90.81%, FMC Corporation, Princetown, New Jersey) in acetone. For each insecticide and the mixtures, five to eight doses were used to provide a range of mortality from 0% to 100%. Two to five day-old, non-blood-fed females of each strain were anaesthetised by extended contact with carbon dioxide and then deposited on a chilled plate (4°C) to maintain anaesthesia during manipulation. Fifty females were used for each dose of insecticide. A volume of 0.1 μl insecticide solution (at the required concentration) was deposited on the upper part of the pronotum of females using a micro-capillary tube. Fifty females that received a volume of 0.1 μl of pure acetone served as controls. After each test, females were transferred to covered plastic cups and provided with a 10% sugar solution on cotton wool and held for 24 hours at 27°C and 80% relative humidity. Mortality rates were recorded 24 hours after the tests. The data were expressed in nanograms of insecticide per milligram of wet female weight and each test was done in triplicate using different batches and generations of mosquitoes.

Data were analysed according to the method of Chou and Talalay [44] using Calcusyn^® ^software [45]. This software provides an accurate estimation of the median-effect doses (analogous to the more familiar LD_50 _values) ± 95% confidence intervals for each insecticide and their mixture. The median effect plot states that:

*Log*(*Fa/Fu*) = *m *× *Log*(*Dx/Dm*)

where *Fa *and *Fu *are the proportions of mosquitoes affected and unaffected, respectively, by the dose *Dx*. *m *represents the slope of the regression line, and *Dm *the dose required to produce the median effect.

For each strain, a binary mixture of permethrin and carbosulfan was used at a constant ratio determined by the ratio of their median-effect doses. At the ratios chosen, both insecticides would be expected to make an equal contribution towards the mortality of each mosquito strain. The existence of interactions between the two insecticides was determined by the Isobologram model of [44], adapted to the analysis of multiple drugs.

## Authors' contributions

Each author contributed towards the design of the experiments. MW, JB, and SD conducted the experiments. CB and PA analyzed the data. CB, PA, MW and VC wrote the paper. All authors read and approved the final manuscript.
